# Measuring Primary Care Spending in the US by State

**DOI:** 10.1001/jamahealthforum.2024.0913

**Published:** 2024-05-17

**Authors:** Deborah J. Cohen, Annette M. Totten, Robert L. Phillips, Yalda Jabbarpour, Jennifer DeVoe

**Affiliations:** 1 Departments of Family Medicine and Medical Informatics and Clinical Epidemiology, School of Medicine, Oregon Health & Science University, Portland; 2 Pacific Northwest Evidence-Based Practice Center, Department of Medical Informatics and Clinical Epidemiology, School of Medicine, Oregon Health & Science University, Portland; 3 The Center for Professionalism and Value in Health Care, American Board of Family Medicine, Washington, DC; 4 Robert Graham Center, Washington, DC; 5 Department of Family Medicine, School of Medicine, Oregon Health & Science University, Portland

## Abstract

This case series identifies states’ estimates of primary care spending and recommends steps policymakers can take toward standardizing these estimates.

## Introduction

Accurately measuring primary care spending is essential to improving health care delivery and outcomes.^1,2^ Herein, we identify states’ estimates of primary care spending and recommend steps policymakers can take toward standardizing these estimates.

## Methods

We conducted searches in Ovid MEDLINE and Cochrane Central from inception to May 2, 2023, as well as the gray literature, to identify state estimates of primary care spending. Methods are described in detail in the Agency for Healthcare Research and Quality Technical Brief No. 44,^3^ including how states measure primary care spending.

## Results

**Figure 1.**
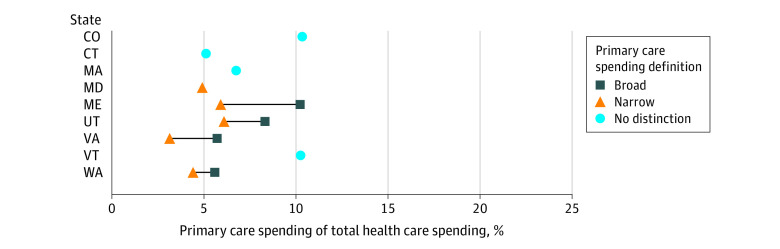
Primary Care Spending as a Percentage of Total Health Care Spending This figure shows primary spending as a percentage of total health care spending for 9 US states. It shows how these estimates vary by state and by definition of primary care (broad, narrow, or no distinction).

Nine states reported estimates of primary care spending as a percentage of total health care spending (Figure 1). Maine, Utah, Virginia, and Washington used different narrow definitions of primary care; their estimates ranged from 3.1% to 6.1% of total spending. Maryland, Maine, Virginia, Utah, and Washington used different broad definitions of primary care; their estimates ranged from 5.6% to 10.2% of total spending. Connecticut, Massachusetts, Vermont, and Colorado did not define primary care as narrow or broad; their estimates ranged from 5.1% to 10.3% of total spending.

**Figure 2.**
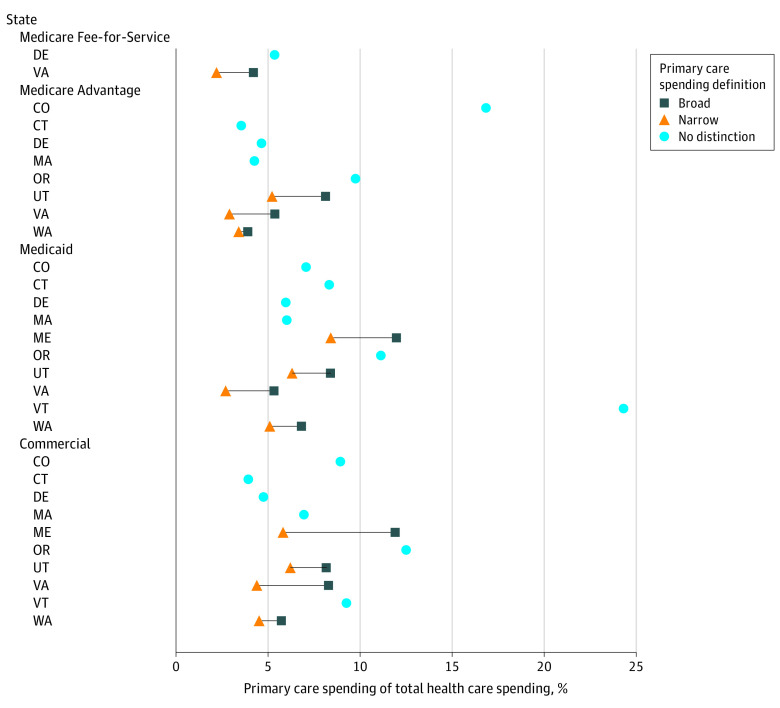
Primary Care Spending Estimates by Payer Type This figure shows primary spending as a percentage of total health care spending by payer type (Medicare Fee-for-Service, Medicare Advantage, Medicaid, or commercial). It shows how these estimates vary by state and by definition of primary care (broad, narrow, or no distinction).

Ten states estimated primary care spending by payer type. All 10 provided estimates for commercial payers, and some provided estimates for Medicaid, Medicare Advantage, and Medicare Fee-for-Service (Figure 2). Oregon, Colorado, Vermont, and Maine reported higher percentages of primary care spending across payer types. Colorado and Oregon included behavioral health clinicians (BHCs) in their numerator (primary care expenditures), and Colorado, Massachusetts, and Oregon did not include prescription drugs in their denominator (total health care expenditures).

## Discussion

In this case series, we identified sizable differences in state primary care spending estimates. We cannot determine if spending actually differs across states, time, or in response to policies because there is no standard method of measurement. This weakens a potentially powerful tool to promote and monitor investment in primary care.

States had similar intentions to base spending estimates on activities of primary care clinicians. States included family medicine, general pediatrics, general internal medicine, and adolescent medicine physicians, as well as nurse practitioners (NPs) and physicians assistants (PAs), as primary care clinicians. Some states also included obstetricians/gynecologists and BHCs. We recommend including just the primary care services delivered by obstetricians/gynecologists; doing so has a small effect on spending estimates.^4^ We recommend including BHCs, if they practice in primary care clinics.

States, however, struggle with reliably identifying the primary care workforce. For example, nationally, it is believed that less than 30% of NPs and 25% of PAs work in primary care.^1^ A federal-state partnership is needed to create and maintain a public primary care database that allows for precise identification of primary care clinics and clinicians. Without this, states default to choosing settings and services that do not accurately differentiate primary care from specialty care for professionals such as BHCs, NPs, and PAs, or even internal medicine physicians who may specialize in addiction or emergency medicine. This approach contributes to wide definitional variance, lack of precision, and inflated primary care spending estimates.^2^

A standardized measure of primary care spending should include all payers and the primary care services provided to all people. It should include claims and nonclaims payments, as well as patient cost sharing and charity care. Total health care spending should include all health care spending; excluding some health care spending, such as prescription drug costs, distorts primary care spending estimates and may contribute to higher primary care spending estimates in some states.^5^

Incomplete or missing data in state reports and lack of clarity and consistency regarding the decisions required to operationalize a measurement of primary care spending limit the ability to make comparison across estimates and to evaluate how differences in estimating decisions might affect the spending estimate.^3^ A standard definition of primary care spending and a transparent way of documenting state-specific decisions is essential for monitoring and improving primary care investment. Standardization will enable policymakers and researchers to understand better how primary care spending is affected by new policies and incentives and, ultimately, how this spending is associated with health outcomes.

## Notes

Supplement. Data Sharing Statement
