# Supplementary material for: Measuring Primary Care Spending in the US by State
Source: JAMA Health Forum. 2024 May 17;5(5):e240913. doi: 10.1001/jamahealthforum.2024.0913 (PMC11102012; doi:10.1001/jamahealthforum.2024.0913)
Supplement: Supplement. — Data Sharing Statement [file jamahealthforum-e240913-s001.pdf]

Data Sharing Statement

Cohen. Measuring Primary Care Spending in the US by State. *JAMA Health Forum*. Published May 17, 2024. doi:10.1001/jamahealthforum.2024.0913

Data
Data available: Yes
Data types: Data (not involving human participants), Other (please specify)
Additional Information: All of the documents that we collected are available in the technical brief.
How to access data: We don't collect any data that involves human subjects.
When available: With publication

Supporting Documents
Document types: Other (please specify)
Additional Information: All documents will be available as part of the release of the technical brief. And, these are publicly available document that we have used in the research letter.
How to access documents: on the AHRQ website. These are also available on state websites.
When available: With publication

Additional Information
Who can access the data: Data is public available to everyone.
Types of analyses: For any purpose.
Mechanisms of data availability: Data are openly available to all.
Any additional restrictions: None
